# Supplementary material for: Deep learning interpretation of echocardiograms
Source: NPJ Digit Med. 2020 Jan 24;3:10. doi: 10.1038/s41746-019-0216-8 (PMC6981156; doi:10.1038/s41746-019-0216-8)
Supplement: Supplementary file 1 — Supplementary Information [file 41746_2019_216_MOESM1_ESM.pdf]

Supplement

A More Details of Data Preparation Methods

Echocardiogram DICOM file pointers and measurements were extracted from the Xcelera database from the Stanford Echocardiography Database. From June 2018 to December 2018, 3312 consecutive full echocardiogram studies (excluding stress echocardiography, transesophageal echocardiograms, limited echocardiogram studies) were downloaded to our research server. All echocardiogram studies were performed using Philips Epiq 7 Cardiology Ultrasound Machines with X5-1 ultrasound probes following a standardized lab protocol for comprehensive echocardiogram studies. Python libraries, including pydicom, CTP, numpy, openCV and scikit were used to anonymize, crop, and downsample the subsequent video files. A combination of neural network based view classification described by Zhang et al as well as using data from the Xcelera database was used to create view specific file datasets for model training.

Training data comprised of 11 frames from each video sampled at 10 frames per second cropped to approximately 700x700 pixels to exclude information outside the scanning sector and then subsequently downsampled to 299x299 pixels with cubic interpolation using the openCV library. Inception-ResNet applies a deep conventional network with residual connections first described by Szegedy et al on an input image of size 299x299 pixels. Each image is passed through a very deep convolutional network with 20 total layers of residual Inception blocks intersperced with batch normalization and reduction layers. Depending on the training task, the final layer after maxpooling underwent a softmax function to generate a probability distribution for which the highest probability class was chosen as the prediction.

B Additional volumetric predictions

Supplementary Figure 1 depicts the Bland-Altman plots for the regression prediction task performance for right atrial major axis length and left atrial volume.

C Summary of EchoNet's Prediction Performance

Supplementary Table 1 summurazies the performance of our method on various tasks.

Prediction task	AUC	f1 score	R^2 score	MAE	
				Ours	Baseline
Sex	0.87	0.82	-	-	-
Age	-	-	0.46	9.8	13.4
Weight (Kg)	-	-	0.56	10.7	15.4
Height (m)	-	-	-	0.07	0.09
Pacemaker or Defibrillator Lead	0.88	0.73	-	-	-
Severe Left Atrial Enlargement	0.86	0.68	-	-	-
Normal Mitral Valve Leaflets	0.75	0.56	-	-	-
End Diastolic Volume (mL)	-	-	0.70	20.5	35.4
End Systolic Volume (mL)	-	-	0.74	13.3	25.4
Ejection Fraction (%)	-	-	0.50	7.0	9.9

Supplementary Table 1. Prediction performance of EchoNet for each prediction task.

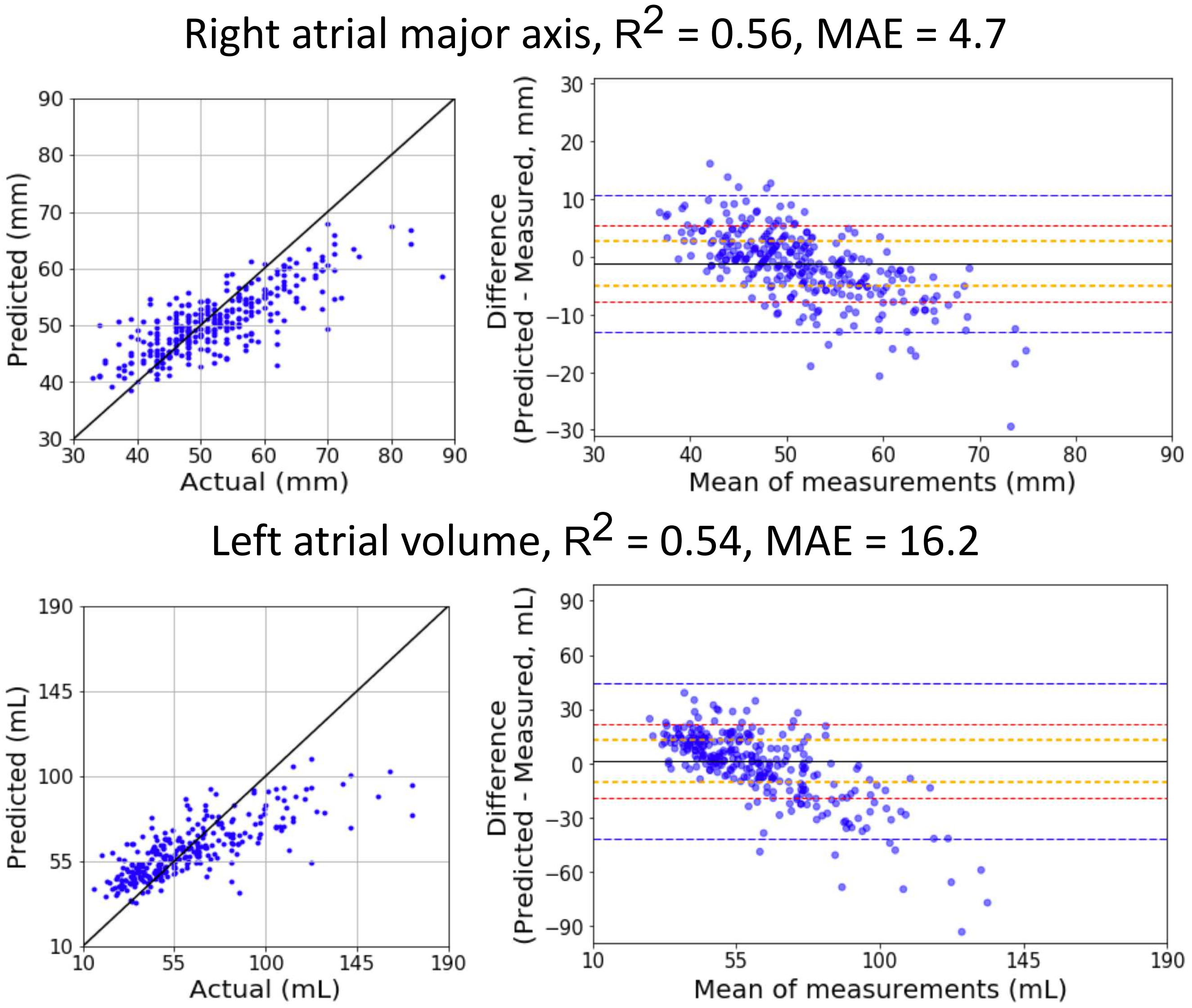

Supplementary Figure 1. Additional linear and volumetric predictions EchoNet performance for prediction of right atrium major axis length and left atrial volume visualized as scatter plots and Bland-Altman plots.
